# Why and how to proceed to an ultrasound-guided transvaginal drainage of tubo-ovarian abscesses (with demonstrative video)?

**DOI:** 10.4274/jtgga.galenos.2019.2019.0131

**Published:** 2020-09-03

**Authors:** Erdogan Nohuz, Géry Lamblin, Clémentine Sciard, Mélodie Mathé, Karine Lebail-Carval, Gautier Chêne

**Affiliations:** 1 Department of Gynecological Surgery, Hôpital Femme Mère Enfant, HFME, Hospices civils de Lyon, Bron, France

**Keywords:** Abscess/surgery, pyosalpingitis, ultrasound-guided, acute salpingitis, adnexal abscess

## Abstract

The treatment of the acute phase of complicated tubo-ovarian abscesses relies on antibiotics associated with surgical management in case of visible abscess, poor clinical tolerance and resistance to medical treatment. Transvaginal, ultrasound-guided, puncture drainage can be considered as an alternative to initial laparoscopy and has multiple advantages over the latter: same success rate, less invasive nature, simple and fast to perform, easy to access, better tolerated by the patient, decreased hospitalization time and less cost. This video article describes and standardizes the essential steps to perform a transvaginal adnexal abscess drainage with a step-by-step explanation of the technique in logical sequences, making the procedure ergonomic and easy to learn. Thus, as part of minimally invasive approach, this technique is henceforth suggested as an effective alternative and signifies a first-line procedure that can promote a therapeutic de-escalation strategy.

## Introduction

The treatment of the acute phase of complicated tubo-ovarian abscesses relies on antibiotics associated with surgical management in case of visible abscess, poor clinical tolerance and resistance to medical treatment (1). The French College of Gynecologists and Obstetricians recommended in 2012 that abscesses greater than 3 cm should be drained by interventional radiology, preferably transvaginally, or laparoscopically, because of the risk of serious complications in the absence of drainage (2). Transvaginal ultrasound-guided drainage in women without signs of acute abdomen or peritonitis and who are clinically stable, should henceforth be considered as an effective alternative, in our opinion. In our experience the transvaginal approach combined with antibiotic therapy is efficient for treatment of tubo-ovarian abscess. This combination promotes the effectiveness of antibiotics, thereby reducing the need for surgical treatment and thus avoiding the potential risks associated with general anesthesia and surgery. In addition this technique contributes to improve the clinical outcome and consequently, to decrease costs and morbidity (3,4). This video article aims to describe and standardize the essential steps to perform a transvaginal adnexal abscess drainage with a step-by-step explanation of the technique in logical sequences, making the procedure easy to learn, ergonomic and safe.

### Technique of ultrasound-guided puncture-drainage

The patient, as welle as the anesthetic and surgical team, should be warned that the puncture may, in certain rare situations, fail and therefore require laparoscopy. In addition, iterative puncture may exceptionally be necessary due to the persistence of residual tubo-ovarian abscess.

This technique offers a direct route from the vaginal wall into areas where tubo-ovarian abscesses are usually situated. The procedure is performed under neuroleptanalgesia, local or general anesthesia after the initiation of broad-spectrum antibiotics. Thus, it can be considered immediately once the abscess has been visually identified, or be delayed from a few hours to a few days depending on the clinical context, to “cool” the lesions and allow antibiotic coverage of the drainage procedure. This involves tri-antibiotic therapy with cephalosporin, cycline and metronidazole, in the absence of allergy, initially administered intravenously (5).

**Figure 1:**
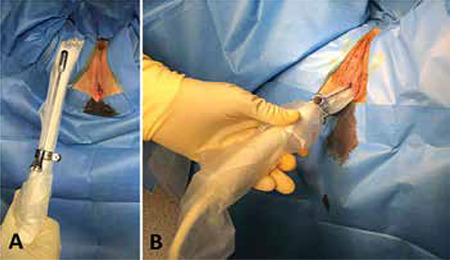
Ultrasound scanning to identify the collection and prepare its puncture. The endovaginal ultrasound probe is covered with a sterile probe cover and gel and equipped with a guide for follicular puncture (A). Ultrasound scanning permits to identify the collection to be evacuated and located immediately in contact with the vaginal wall (B).

**Figure 2:**
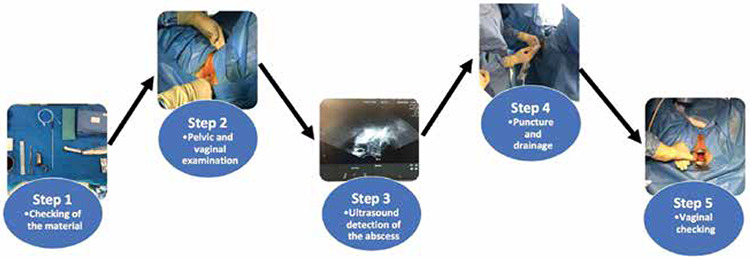
Main steps to perform an ultrasound-guided transvaginal puncture with aspirative drainage. (Step 1) Checking of the material. An endovaginal transducer with an attached biopsy guidance system and needle with a connection tube and syringe are used. (Step 2) Physical examination (vaginal palpation). (Step 3) Ultrasound detection of the abscess and setting of the shooting window. (Step 4) Transvaginal puncture next to the abscess, and drainage-aspiration under ultrasound control. (Step 5) Vaginal checking and cleaning. The aspirated fluid is sent for microbiological analysis.

**Figure 3:**
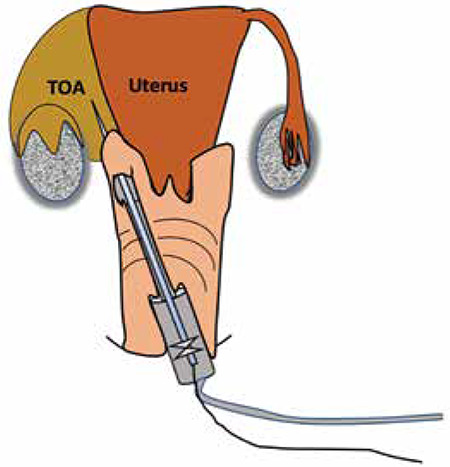
Diagram showing puncture-drainage. Note the proximity of the vaginal cul-de-sac with the tubo-ovarian abscess TOA: Tubo-ovarian abscess

The patient is placed in a gynecological position. Vaginal cleaning with povidone-iodine preparation is performed to ensure a sterile field. The bladder should be positively identified using ultrasound which is facilitated by absence of prior urinary catheterization makes it possible. This is important to avoid bladder injury and to have a fixed anatomical landmark. If necessary, partial bladder emptying will limit the risk of bladder injury. Palpation of the abscess should be attempted which aids in orientation of the ultrasound probe during the puncture’ step. An endovaginal ultrasound probe (6 to 10 MHz frequency), covered with a sterile probe cover and gel, is equipped with a guide for follicular punctures (Figure 1a). Ultrasound scanning is then performed to identify the abscess and prepare its puncture (Figure 1b). Once the abscess is visualized, which must be located immediately in contact with the vaginal wall in order to avoid any digestive transfixion, a puncture is performed under permanent ultrasound control (Figure 2,3 and Video 1). We use a 17 gauge/1.5 mm puncture-aspiration bevel needle, the distal end of which is echogenic, to follow the path and facilitate the procedure. A syringe is screwed to the tube connected to the needle. The use of an automatic follicular suction pump is ergonomic and facilitates aspiration. If one is dealing with a collection of thick consistency, it may sometimes be useful to inject saline. A catheter can be left in place to permit further drainage. However, this possibility carries the risk of the catheter becoming displaced. There is no evidence as to the benefit of leaving a catheter in situ and thus we do not recommend this. At the end of the puncture-drainage, a speculum examination of the vagina and an antiseptic cleaning ends the procedure. The sample is sent for bacteriological analysis in order to possibly adjust antibiotic treatment to identified organisms and sensitivities.

The limits of this technique lie in the inability to identify a possible adnexal malignant lesion and the impossibility of evaluating the tubal state in patients with a desire for pregnancy, although a laparoscopy performed a few months later and under better surgical conditions, allows the treatment of possible sequelae such as adhesions and/or tubal stenosis. Moreover, this approach has not yet been compared to that of laparoscopy by clinical study. Thus, a French multicenter randomized trial is currently under way. The PACTOL study aims to demonstrate that the transvaginal method is no less effective than laparoscopy to treat tubo-ovarian abscesses. This study should be able to answer questions that are still outstanding concerning the use of this technique such as impact on chronic pelvic pain and future fertility (5).

## Conclusion

**Figure 4:**
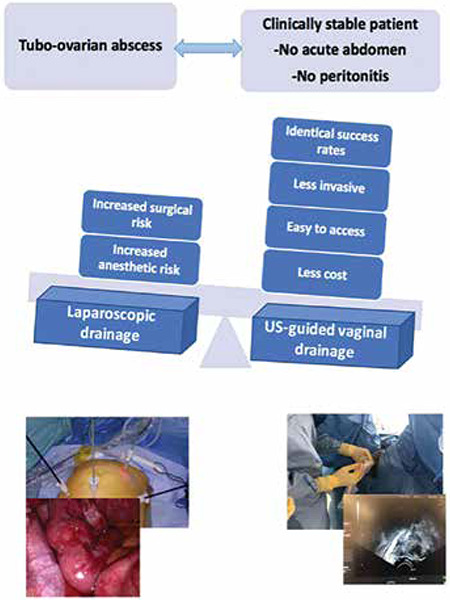
Graphical abstract. Comparison between laparoscopic and transvaginal approaches of adnexal abscesses US: Ultrasound

Ultrasound-guided, transvaginal drainage is an alternative to initial laparoscopic drainage of tubo-ovarian abscesses. We believe that it should replace laparoscopic drainage because of multiple advantages. These include identical success rate and being less invasive. Laparoscopy or laparotomy may in some cases prove to be complex and a source of intestinal wounds, whether it be a conservative surgery or an excisional procedure. In addition transvaginal drainage is simple and fast to perform, normally taking only 15-20 minimum, is easy to access, is better tolerated by the patient, and results in decreased hospitalization time and less cost (Figure 4, graphical abstract). Thus, as part of a minimally invasive approach, it represents a first-line procedure that can promote the therapeutic de-escalation strategy.

***Video 1. https://www.doi.org/10.4274/jtgga.galenos.2019.2019.0131.video1***
